# Mapping Quantitative Trait Loci Using Distorted Markers

**DOI:** 10.1155/2009/410825

**Published:** 2010-02-21

**Authors:** Shizhong Xu, Zhiqiu Hu

**Affiliations:** Department of Botany and Plant Sciences, University of California, Riverside, CA 92521, USA

## Abstract

Quantitative trait locus (QTL) mapping is usually performed using markers that follow a Mendelian segregation ratio. We developed a new method of QTL mapping that can use markers with segregation distortion (non-Mendelian markers). An EM (expectation-maximization) algorithm is used to estimate QTL and SDL (segregation distortion loci) parameters. The joint analysis of QTL and SDL is particularly useful for selective genotyping. Application of the joint analysis is demonstrated using a real life data from a wheat QTL mapping experiment.

## 1. Introduction

Segregation distortion is a phenomenon that the genotypic frequency array of a locus does not follow a typical Mendelian ratio. Depending on the population under investigation, Mendelian ratio of a locus varies from 1 : 1 for a backcross to 1 : 2 : 1 for an F_2_ and to 1 : 1 : 1 : 1 for a four-way cross. These ratios hold for codominant markers. For some reasons, a marker may not follow a typical Mendelian ratio. Such a marker is called a distorted marker. For a long period of time, the effects of distorted markers on the result of QTL mapping were not known. For the reason of precaution, people simply discarded all the distorted markers in QTL mapping. Recently, we found that distorted markers can be safely used for QTL mapping with no detrimental effect on the result of QTL mapping [1]. This finding can help QTL mappers save tremendous resources by using all available markers, regardless whether they are Mendelian or not. We also found that if distorted markers are handled properly, they can be beneficial to QTL mapping. 

Marker segregation distortion is only a phenomenon. The reason behind the distortion is due to one or more segregation distortion loci (SDL). These loci are subject to gametic selection [2], zygotic selection [3], or both and their (unobservable) distorted segregation causes the observed markers to deviate from the Mendelian ratio. Several investigators [4–11] have attempted to map these segregation distortion loci using molecular markers. It is natural to consider mapping QTL and SDL jointly in the same population. Agricultural scientists are interested in mapping QTL for economically important traits while evolutionary biologists are interested in mapping SDL that respond to natural selection. Combining the two mapping strategies into one is beneficial to both communities. Performing such a joint mapping strategy is the main objective of this study. Since the theory of segregation distortion has been introduced and discussed in previous studies [7, 8] and our own research [1], this study only presents the EM (expectation-maximization) implementation of the statistical method. The variance-covariance matrix of estimated parameters under the EM algorithm is also derived and presented in Appendix A for interested readers. 

## 2. Methods

We only investigate interval mapping where a model contains a single QTL at a time and the entire genome is scanned through repeated calling of the same program for different locations of the genome. The technical difference between the joint mapping and QTL mapping occurs only in one place. In the traditional interval mapping of QTL, the conditional probabilities of genotypes for a QTL are calculated using flanking marker genotypes with the prior probabilities of QTL genotypes being substituted by the Mendelian ratio. For the joint mapping, the genotypic frequencies (segregation ratios) are treated as unknown parameters that are subject to estimation. We use an F_2_ population as an example to demonstrate the method. Extension to other population is discussed subsequently. 

### 2.1. The Likelihood of Markers

Let *M* and *N* be the left and right flanking markers bracketing the QTL (denoted by *G* for short). The interval of the genome carrying the three loci is labeled by a segment *M* *G* *N*. The marker linkage phases are known for line crosses derived from inbred lines. The three genotypes of the QTL are denoted by *G*_1_ *G*_1_, *G*_1_ *G*_2_, and *G*_2_ *G*_2_, respectively. Similar notation also applies to the genotypes of the flanking markers. The interval defined by markers *M* and *N* is divided into two segments. Let *r*_1_ and *r*_2_ be the recombination fractions for segments *M* *G* and *G* *N*, respectively. The joint distribution of the marker genotypes conditional on the QTL genotype can be derived using the Markov chain property under the assumption of no interference between consecutive loci in segregation. Let us denote the three ordered genotypes, *G*_1_ *G*_1_, *G*_1_ *G*_2_, and *G*_2_ *G*_2_, by genotypes 1, 2, and 3, respectively. If individual *j* takes the *κ*th genotype for the QTL, we denote the event by *G*_*j*_ = *κ*, for all *κ* = 1, 2, 3. The joint probability of the two markers conditional on the genotype of the QTL is

(1) $$\begin{array}{cc} & \text{Pr}\left(M_{j}=\xi ,N_{j}=\zeta \;|\;G_{j}=\kappa \right)\\ & \;\;=\text{Pr}\left(M_{j}=\xi \;|\;G_{j}=\kappa \right)\text{Pr}\left(N_{j}=\zeta \;|\;G_{j}=\kappa \right) \end{array}$$

for all *κ*, *ξ*, *ζ* = 1, 2, 3, where Pr(*M*_*j*_ = *ξ* ∣ *G*_*j*_ = *κ*) = Γ_1_(*κ*, *ξ*) and Pr(*N*_*j*_ = *ζ* ∣ *G*_*j*_ = *κ*) = Γ_2_(*κ*, *ζ*). We use Γ_*i*_(*κ*, *ξ*) to denote the *κ*th row and the *ξ*th column of the following transition matrix:

(2) $$\Gamma_{i}=\left[\begin{array}{ccc} {\left(1-r_{i}\right)}^{2} & 2r_{i}\left(1-r_{i}\right) & r_{i}^{2}\\ r_{i}\left(1-r_{i}\right) & {\left(1-r_{i}\right)}^{2}+r_{i}^{2} & r_{i}\left(1-r_{i}\right)\\ r_{i}^{2} & 2r_{i}\left(1-r_{i}\right) & {\left(1-r_{i}\right)}^{2} \end{array}\right],\;\forall i=1,2.$$

For example,

(3) $$\begin{array}{cc} & \text{Pr}\left(M_{j}=1,N_{j}=2\;|\;G_{j}=3\right)\\ & \;\;=\text{Pr}\left(M_{j}=1\;|\;G_{j}=3\right)\text{Pr}\left(N_{j}=2\;|\;G_{j}=3\right)\\ & \;\;=\Gamma_{1}\left(3,1\right)\Gamma_{2}\left(3,2\right)=2r_{1}^{2}r_{2}\left(1-r_{2}\right). \end{array}$$

Let *ω*_*κ*_ = Pr(*G* = *κ*), for all *κ* = 1, 2, 3, be the probability that a randomly sampled individual from the F_2_ family has a genotype *κ*. We use a generic notation *p* for probability so that *p*(*G*_*j*_ = *κ*) represents Pr(*G*_*j*_ = *κ*) and *p*(*M*_*j*_, *N*_*j*_ ∣ *G*_*j*_ = *κ*) stands for Pr(*M*_*j*_, *N*_*j*_ ∣ *G*_*j*_ = *κ*). The log likelihood function of the flanking marker genotypes in the F_2_ population is

(4) $$\begin{array}{cc} L\left(\omega \;|\;m\right) & =\overset{n}{\underset{j=1}{\sum }}\ln\;\left[\overset{3}{\underset{\kappa =1}{\sum }}p\left(G_{j}=\kappa \right)p\left(M_{j},N_{j}\;|\;G_{j}=\kappa \right)\right]\\ & =\overset{n}{\underset{j=1}{\sum }}\ln\;\left[\overset{3}{\underset{\kappa =1}{\sum }}\omega_{\kappa }\Gamma_{1}\left(\kappa ,M_{j}\right)\Gamma_{2}\left(\kappa ,N_{j}\right)\right], \end{array}$$

where *ω* ^T^ = [*ω*_1_ *ω*_2_ *ω*_3_] is a vector of parameters with constraint ∑_*κ*=1_^3^ *ω*_*κ*_ = 1, where *m* in *L*(*ω* ∣ *m*) stands for marker information. Note that without any prior information, *p*(*G*_*j*_ = *κ*) = *ω*_*κ*_, for all *j* = 1,…, *n*. Under the assumption of Mendelian segregation, *ω* = *ϕ* where *ϕ* = [*ϕ*_1_ *ϕ*_2_ *ϕ*_3_]^T^ = [1/4  1/2  1/4]^T^. However, we treat *ω* as unknown parameters. Because we are dealing with the genotypic frequencies, the segregation distortion is called zygotic distortion. Segregation distortion due to gametic selection will be discussed later. We postulate that deviation of *ω* from *ϕ* causes a marker linked to locus *G* to show distorted segregation. This likelihood function is the one used in mapping viability loci [10].

### 2.2. The Likelihood of Phenotypes

Let *y*_*j*_ be the phenotypic value of a quantitative trait measured from individual *j*. The probability density of *y*_*j*_ conditional on the genotype of individual *j* is normal with mean

(5) $$\mu_{\kappa }=X_{j}\beta +H_{\kappa }\gamma$$

and variance *σ* ^2^, that is,

(6) $$p\left(y_{j}\;|\;G_{j}=\kappa \right)=\frac{1}{\sqrt{2\pi }\sigma^{2}}\exp\;\left[-\frac{1}{2\sigma^{2}}{\left(y_{j}-X_{j}\beta -H_{\kappa }\gamma \right)}^{2}\right],$$

where *H*_*κ*_ is the *κ*th row of matrix *H* and

(7) $$H=\left[\begin{array}{cc} +1 & -1\\ 0 & 1\\ -1 & -1 \end{array}\right].$$

This *H* matrix can be defined in a different scale, for example,

(8) $$H=\left[\begin{array}{cc} +1 & 0\\ 0 & 1\\ -1 & 0 \end{array}\right],$$

which does not affect the significance test. The advantage of choosing the scale in (7) is that the expectation of the dominance indicator is zero. Vector *γ* = [*a* *d*]^*T*^ contains the additive and dominance effects. The design matrix *X*_*j*_ and the regression coefficients *β* capture non-QTL effects, for example, field location effects, year effects, and so on. The likelihood function of the phenotypic values in the F_2_ population is

(9) $$\begin{array}{cc} & L\left(\beta ,\gamma ,\sigma^{2},\omega \;|\;y\right)\\ & \;\;=\overset{n}{\underset{j=1}{\sum }}\ln\;\left[\overset{3}{\underset{\kappa =1}{\sum }}p\left(G_{j}=\kappa \right)p\left(y_{j}\;|\;G_{j}=\kappa \right)\right]\\ & \;\;=-\frac{n}{2}\ln\;\left(\sigma^{2}\right)+\overset{n}{\underset{j=1}{\sum }}\ln\;\left\{\overset{3}{\underset{\kappa =1}{\sum }}\omega_{\kappa }\exp\;\left[-\frac{1}{2\sigma^{2}}{\left(y_{j}-\mu_{\kappa }\right)}^{2}\right]\right\}, \end{array}$$

where letter *y* in *L*(*β*, *γ*, *σ* ^2^, *ω* ∣ *y*) stands for the phenotype. This likelihood function is the one used in segregation analysis of quantitative traits [12] because no marker information is required.

### 2.3. Joint Likelihood of Markers and Phenotypes

Let *θ* = [*β* ^*T*^ *γ* ^*T*^ *σ* ^2^ *ω* ^T^]^*T*^ be a vector of all parameters in the joint analysis. The likelihood function is obtained by combining (4) and (9):

(10) $$\begin{array}{cc} & L\left(\theta \;|\;m,y\right)\\ & =\overset{n}{\underset{j=1}{\sum }}\ln\;\left[\overset{3}{\underset{\kappa =1}{\sum }}p\left(G_{j}=\kappa \right)p\left(y_{j}\;|\;G_{j}=\kappa \right)p\left(M_{j},N_{j}\;|\;G_{j}=\kappa \right)\right]\\ & =\overset{n}{\underset{j=1}{\sum }}\ln\;\left\{\overset{3}{\underset{\kappa =1}{\sum }}\omega_{\kappa }\exp\;\left[-\frac{1}{2\sigma^{2}}{\left(y_{j}-\mu_{\kappa }\right)}^{2}\right]\Gamma_{1}\left(\kappa ,M_{j}\right)\Gamma_{2}\left(\kappa ,N_{j}\right)\right\}\\ & \;-\frac{n}{2}\ln\;\left(\sigma^{2}\right). \end{array}$$

For QTL mapping under segregation distortion, this log likelihood function is the one to be maximized. The previous two likelihood functions (for markers and for phenotypes) were presented as background information to introduce this joint log likelihood function.

### 2.4. EM Algorithm for the Joint Analysis

The MLE (maximum likelihood estimate) of the parameters is solved via an EM algorithm [13]. We need to rewrite the likelihood function in a form of complete data. Let us define a delta function as

(11) $$\delta \left(G_{j},\kappa \right)=\{\begin{array}{cc} 1 & \text{if }G_{j}=\kappa ,\\ 0 & \text{if }G_{j}\neq \kappa , \end{array}$$

If the genotypes of all individuals are known, that is, given *δ*(*G*_*j*_, *κ*) for all *j* = 1,…, *n* and *κ* = 1, 2, 3, the complete-data log likelihood is

(12) $$L\left(\theta ,\delta \right)=\overset{n}{\underset{j=1}{\sum }}\ln\;\left[p\left(y_{j}\;|\;G_{j}\right)p\left(M_{j},N_{j}\;|\;G_{j}\right)p\left(G_{j}\right)\right],$$

where

(13) $$\begin{array}{cc} p\left(y_{j}\;|\;G_{j}\right) & =\frac{1}{\sqrt{2\pi }\sigma^{2}}\exp\;\left[-\frac{1}{2\sigma^{2}}\overset{3}{\underset{\kappa =1}{\sum }}\delta \left(G_{j},\kappa \right){\left(y_{j}-\mu_{\kappa }\right)}^{2}\right],\\ p\left(M_{j},N_{j}\;|\;G_{j}\right) & =\overset{3}{\underset{k=1}{\prod }}p{\left(M_{j},N_{j}\;|\;G_{j}=\kappa \right)}^{\delta (G_{j},\kappa )}\\ & =\overset{3}{\underset{k=1}{\prod }}{\left[\Gamma_{1}(\kappa ,M_{j})\Gamma_{2}(\kappa ,N_{j})\right]}^{\delta (G_{j},\kappa )},\\ p\left(G_{j}\right) & =\overset{3}{\underset{\kappa =1}{\prod }}\omega_{\kappa }^{\delta (G_{j},\kappa )}. \end{array}$$

The delta variables are missing values. Therefore, we need to take expectation of the log likelihood with respect to *δ*. The expected complete-data log likelihood function is

(14) $$L\left(\theta \;|\;\theta^{\left(t\right)}\right)=E_{\delta }\left[L\left(\theta ,\delta \right)\;|\;\theta^{\left(t\right)}\right]=\psi_{0}+\psi_{1}\left(\theta \right)+\psi_{2}\left(\theta \right).$$

Note that *E*_*δ*_[*L*(*θ*, *δ*) ∣ *θ* ^(*t*)^] stands for the expectation of *L*(*θ*, *δ*) with respect to *δ* conditional on parameters at the current state *θ* ^(*t*)^ and the data (the symbol for data is suppressed for simplicity). The three components of (14) are

(15) $$\begin{array}{cc} \psi_{0} & =\overset{n}{\underset{j=1}{\sum }}\;\overset{3}{\underset{\kappa =1}{\sum }}E_{\delta }\left[\delta \left(G_{j},\kappa \right)\;|\;\theta^{\left(t\right)}\right]\\ & \;\;\;\;\times \left[\ln\;\Gamma_{1}\left(\kappa ,M_{j}\right)+\ln\;\Gamma_{2}\left(\kappa ,N_{j}\right)\right],\\ \psi_{1}\left(\theta \right) & =-\frac{n}{2}\ln\;\left(\sigma^{2}\right)\\ & \;-\frac{1}{2\sigma^{2}}\overset{n}{\underset{j=1}{\sum }}\;\overset{3}{\underset{\kappa =1}{\sum }}E_{\delta }\left[\delta \left(G_{j},\kappa \right)\;|\;\theta^{\left(t\right)}\right]{\left(y_{j}-\mu_{\kappa }\right)}^{2},\\ \psi_{2}\left(\theta \right) & =\overset{n}{\underset{j=1}{\sum }}\;\overset{3}{\underset{\kappa =1}{\sum }}E_{\delta }\left[\delta \left(G_{j},\kappa \right)\;|\;\theta^{\left(t\right)}\right]\ln\;\omega_{\kappa }. \end{array}$$

The first component *ψ*_0_ is a function of *θ* ^(*t*)^ but not a function of *θ*. Therefore, it is considered as a constant.

#### 2.4.1. Expectation (E-Step)

The expectation step of the EM algorithm requires computing the expectation of *δ* conditional on the data and *θ* ^(*t*)^. Because *δ* is a Bernoulli variable, the expectation is simply the probability of *δ* = 1, that is,

(16) $$\begin{array}{cc} & E_{\delta }\left[\delta \left(G_{j},\kappa \right)\;|\;\theta^{\left(t\right)}\right]\\ & =\text{Pr}\left[\delta \left(G_{j},\kappa \right)=1\;|\;\theta^{\left(t\right)},m,y\right]\\ & =\frac{p\left(G_{j}=\kappa \right)p\left(y_{j}\;|\;G_{j}=\kappa \right)p\left(M_{j},N_{j}\;|\;G_{j}=\kappa \right)}{\sum_{\xi =1}^{3}p\left(G_{j}=\xi \right)p\left(y_{j}\;|\;G_{j}=\xi \right)p\left(M_{j},N_{j}\;|\;G_{j}=\xi \right)}\\ & =\frac{\omega_{\kappa }\exp\;\left[-\left(1/2\sigma^{2}\right){\left(y_{j}-\mu_{\kappa }\right)}^{2}\right]\Gamma_{1}\left(\kappa ,M_{j}\right)\Gamma_{2}\left(\kappa ,N_{j}\right)}{\sum_{\xi =1}^{3}\omega_{\xi }\exp\;\left[-\left(1/2\sigma^{2}\right){\left(y_{j}-\mu_{\xi }\right)}^{2}\right]\Gamma_{1}\left(\xi ,M_{j}\right)\Gamma_{2}\left(\xi ,N_{j}\right)}. \end{array}$$

#### 2.4.2. Maximization (M-Step)

The maximization step of the EM algorithm requires taking the partial derivatives of *L*(*θ* ∣ *θ* ^(*t*)^) with respect to *θ*, setting the partial derivatives equal to zero, and solving for the parameters:

(17) $$\frac{\partial }{\partial \theta }L\left(\theta \;|\;\theta^{\left(t\right)}\right)=\frac{\partial }{\partial \theta }\psi_{1}\left(\theta \right)+\frac{\partial }{\partial \theta }\psi_{2}\left(\theta \right)=0.$$

The solutions of the parameters are

(18) $$\begin{array}{cc} \beta & ={\left[\overset{n}{\underset{j=1}{\sum }}X_{j}^{T}X_{j}^{}\right]}^{-1}\left[\overset{n}{\underset{j=1}{\sum }}\overset{3}{\underset{\kappa =1}{\sum }}E\left[\delta \left(G_{j},\kappa \right)\right]X_{j}^{T}\left(y_{j}-H_{\kappa }\gamma \right)\right],\\ \gamma & ={\left[\overset{n}{\underset{j=1}{\sum }}\;\overset{3}{\underset{\kappa =1}{\sum }}E\left[\delta (G_{j},\kappa )\right]\left(H_{\kappa }^{T}H_{\kappa }^{}\right)\right]}^{-1}\\ & \;\times \left[\overset{n}{\underset{j=1}{\sum }}\;\overset{3}{\underset{\kappa =1}{\sum }}E\left[\delta \left(G_{j},\kappa \right)\right]H_{\kappa }^{T}\left(y_{j}-X_{j}\beta \right)\right],\\ \sigma^{2} & =\frac{1}{n}\overset{n}{\underset{j=1}{\sum }}\;\overset{3}{\underset{\kappa =1}{\sum }}E\left[\delta \left(G_{j},\kappa \right)\right]{\left(y_{j}-X_{j}\beta -H_{\kappa }\gamma \right)}^{2},\\ \omega_{\kappa } & =\frac{1}{n}\overset{n}{\underset{j=1}{\sum }}E\left[\delta \left(G_{j},\kappa \right)\right],\;\forall \kappa =1,2,3. \end{array}$$

### 2.5. Hypothesis Tests

Hypothesis tests are complicated when QTL segregate in a non-Mendelian fashion. There are many different hypotheses we can test here. Although the Wald test can be performed for testing the presence of QTL, such a test is not justified for testing the null hypothesis of Mendelian segregation. Therefore, the likelihood ratio tests are more justifiable. Regardless what hypothesis is tested, the full model joint log likelihood function given in (10) is required. Let us reintroduce this joint log likelihood function using a different notation so that different likelihood ratio tests are easily interpreted. The joint likelihood is rewritten as

(19) $$\begin{array}{cc} & L_{QS}\left(\gamma ,\omega \right)\\ & =-\frac{n}{2}\log\;\left(\sigma^{2}\right)\\ & +\overset{n}{\underset{j=1}{\sum }}\ln\;\left\{\overset{3}{\underset{\kappa =1}{\sum }}\omega_{\kappa }\exp\;\left[-\frac{1}{2\sigma^{2}}{\left(y_{j}-\mu_{\kappa }\right)}^{2}\right]\Gamma_{1}\left(\kappa ,M_{j}\right)\Gamma_{2}\left(\kappa ,N_{j}\right)\right\}, \end{array}$$

where *γ* represents QTL effects and *ω* stands for non-Mendelian segregation. The null hypothesis for QTL detection is *H*_QTL_ : *γ* = 0 while the null hypothesis for detecting segregation distortion is *H*_SDL_ : *ω* = *ϕ*.

#### 2.5.1. Testing the Presence of QTL

The null hypothesis is *H*_QTL_ : *γ* = 0. The likelihood ratio test statistic is

(20) $$\lambda_{\text{QTL}}=-2\left[L_{S}\left(0,\hat{\omega }\right)-L_{QS}\left(\hat{\gamma },\hat{\omega }\right)\right],$$

where $L_{S}(0,\hat{\omega })$ is the log likelihood value under the null model *γ* = 0, which is

(21) $$L_{S}\left(0,\hat{\omega }\right)=L\left(\hat{\beta },{\hat{\sigma }}^{2}\;|\;y\right)+L\left(\hat{\omega }\;|\;m\right),$$

where

(22) $$\begin{array}{cc} L\left(\hat{\beta },{\hat{\sigma }}^{2}\;|\;y\right) & =-\frac{n}{2}\ln\;\left({\hat{\sigma }}^{2}\right)-\frac{1}{2{\hat{\sigma }}^{2}}\overset{n}{\underset{j=1}{\sum }}{\left(y_{j}-X_{j}\hat{\beta }\right)}^{2},\\ L\left(\hat{\omega }\;|\;m\right) & =\overset{n}{\underset{j=1}{\sum }}\ln\;\left[\overset{3}{\underset{\kappa =1}{\sum }}{\hat{\omega }}_{\kappa }\Gamma_{1}\left(\kappa ,M_{j}\right)\Gamma_{2}\left(\kappa ,N_{j}\right)\right]. \end{array}$$

The estimated parameters in (22) are obtained by maximizing the corresponding likelihood functions (see Appendix B for the estimation).

#### 2.5.2. Testing Non-Mendelian Segregation

The null hypothesis is *H*_SDL_ : *ω* = *ϕ*. The likelihood ratio test statistic is

(23) $$\lambda_{\text{SDL}}=-2\left[L_{Q}\left(\hat{\gamma },\phi \right)-L_{QS}\left(\hat{\gamma },\hat{\omega }\right)\right],$$

where

(24) $$\begin{array}{cc} & L_{Q}\left(\hat{\gamma },\phi \right)\\ & =-\frac{n}{2}\ln\;\left({\hat{\sigma }}^{2}\right)\\ & +\overset{n}{\underset{j=1}{\sum }}\ln\;\left\{\overset{3}{\underset{\kappa =1}{\sum }}\phi_{\kappa }\exp\;\left[-\frac{1}{2{\hat{\sigma }}^{2}}{\left(y_{j}-{\hat{\mu }}_{\kappa }\right)}^{2}\right]\Gamma_{1}\left(\kappa ,M_{j}\right)\Gamma_{2}\left(\kappa ,N_{j}\right)\right\}. \end{array}$$

Again, the MLEs of the parameters in (24) are obtained by maximizing this likelihood function (see Appendix B for the estimation of parameters under the null model).

#### 2.5.3. Testing Both QTL and SDL

The null hypothesis is *H*_0_ : *γ* = 0 and *ω* = *ϕ*. The likelihood ratio test statistic is

(25) $$\lambda_{QS}=-2\left[L\left(0,\phi \right)-L_{QS}\left(\hat{\gamma },\hat{\omega }\right)\right],$$

where

(26) $$L\left(0,\phi \right)=L\left(\hat{\beta },{\hat{\sigma }}^{2}\;|\;y\right)+L\left(\phi \;|\;m\right).$$

The two components of (26) are

(27) $$\begin{array}{cc} L\left(\hat{\beta },{\hat{\sigma }}^{2}\;|\;y\right) & =-\frac{n}{2}\ln\;\left({\hat{\sigma }}^{2}\right)-\frac{1}{2{\hat{\sigma }}^{2}}\overset{n}{\underset{j=1}{\sum }}{\left(y_{j}-X_{j}\hat{\beta }\right)}^{2},\\ L\left(\phi \;|\;m\right) & =\overset{n}{\underset{j=1}{\sum }}\ln\;\left[\overset{3}{\underset{\kappa =1}{\sum }}\phi_{\kappa }\Gamma_{1}\left(\kappa ,M_{j}\right)\Gamma_{2}\left(\kappa ,N_{j}\right)\right]. \end{array}$$

The parameters involved in these log likelihood functions are estimated using formulas given in Appendix B. This hypothesis is rejected if either *γ* ≠ 0 or *ω* ≠ *ϕ* or both inequalities hold. The QTL effects and the segregation distortion are confounded. This hypothesis test is useful in the following situation. Suppose that, for some reason, we know for sure that the population from which the sample is drawn is a Mendelian population. The sample drawn from this population is selected based on extreme phenotypes (selective genotyping). The sample is then non-Mendelian regarding the QTL that control the trait subject to phenotypic selection. Rejecting this hypothesis is equivalent to rejecting the null hypothesis of QTL. The reason is that segregation distortion in the sample is solely caused by selective genotyping. Therefore, this joint test can be used to detect QTL under selective genotyping.

## 3. Applications

This example demonstrates the application of the method to joint mapping of QTL and SDL in a wheat QTL mapping experiment. The experiment was conducted by Dou et al. [14] who made the data available to us for this analysis. A female sterile line XND126 and an elite cultivar Gaocheng 8901 with normal fertility were crossed for genetic analysis of female sterility measured as a ratio of the number of seeded spikelets to the total number of spikelets per plant. The parents and their F_1_ and F_2_ progeny were planted in the Huaian experimental station in China for the 2006-2007 growing season under the normal autumn sowing condition. The mapping population was the F_2_ family consisting of 234 individual plants. The sterility trait was transformed using the angular sine transformation, *y* = arcsin(*x*), where *x* is the phenotypic value expressed as ratio. A total of 28 SSR markers were used in this experiment. These markers covered five chromosomes of the wheat genome with an average genome marker density of 15.5 cM per marker interval. The five chromosomes are only part of the wheat genome. The model for the female sterility is

(28) $$y_{j}=X_{j}\beta +Z_{j1}\gamma_{1}+Z_{j2}\gamma_{2}+\varepsilon_{j},$$

where *X*_*j*_ = 1 for all *j*, *β* is the intercept, *Z*_*j*1_ = {+1,0, − 1} is the genotype indicator variable for the additive effect *γ*_1_ = *a*, and *Z*_*j*2_ = {-1,1, − 1} is the genotype indicator variable for the dominance effect *γ*_2_ = *d*. We used an interval mapping approach to scanning the entire genome. Therefore, the model contains one QTL at a time. With the interval mapping, *Z*_*j*_ = [*Z*_*j*1_ *Z*_*j*2_] is missing and can take one of three values denoted by *H*_*κ*_ for *κ* = 1, 2, 3 (see definition of *H*_*κ*_) in Section 2. 

**Figure 1:**
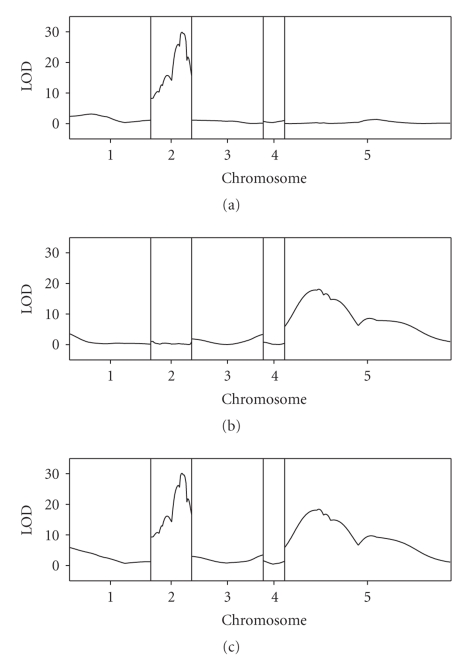
LOD score profiles for the wheat genome. The 5 chromosomes of the genome are separated by the gray reference lines. (a) The top panel represents the LOD profile for testing significance of QTL for the female sterility of wheat (regardless whether segregation is distorted or not). (b) The panel in the middle represents the LOD profile for testing significance of SDL (regardless whether a QTL is present or not). (c) The panel at the bottom represents the LOD profile for testing both QTL and SDL (joint test and the null model being no QTL and no SDL).

**Figure 2:**
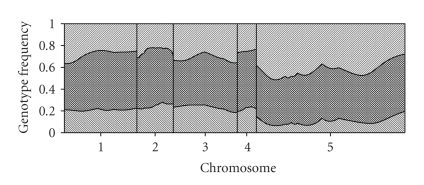
Estimated genotypic frequencies for the wheat genome. Frequencies of the three genotypes are represented by areas with different patterns. Chromosomes are separated by the gray reference lines.

**Figure 3:**
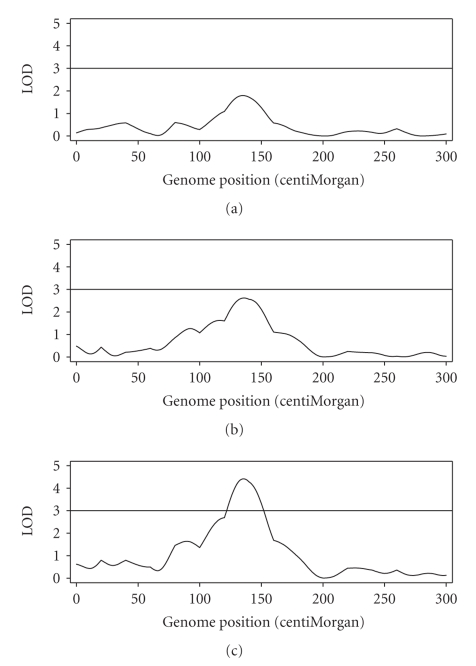
LOD score profiles for the simulated genome (single chromosome). The horizontal line at LOD = 3 represents the threshold. (a) The top panel represents the LOD profile for testing significance of QTL for the simulated trait (regardless whether segregation is distorted or not). (b) The panel in the middle represents the LOD profile for testing significance of SDL (regardless whether a QTL is present or not). (c) The panel at the bottom represents the LOD profile for testing both QTL and SDL (joint test and the null model being no QTL and no SDL).

The likelihood ratio test statistics were divided by 4.61 to obtain their corresponding LOD scores. The LOD score profiles across the genome are shown in Figure 1. The top panel (a) shows the LOD profile for QTL detection regardless whether there is segregation distortion or not. One major QTL was detected in the second chromosome. This chromosome segregates normally without distortion. Figure 3(b) shows the LOD profile for testing segregation distortion, regardless whether the QTL is present or absent. One major SDL was found on chromosome five. Figure 3(c) is the joint LOD score for both QTL and SDL. We can see that both the major QTL and the SDL were detected. These two major loci have very high LOD scores. We used the quick method of Piepho [15] to calculate the genome wide critical value of the LOD for significance test. We found that the genome wide critical value was slightly less than LOD = 3 criterion (data not shown). Therefore, we set LOD = 3 as the criterion. In addition to the two major loci, several other regions of the genome also showed significant peaks of the LOD score profile. The estimated parameters are listed in Table 1. Overall we detected eight loci, four QTLs and four SDLs. Among the detected QTL, each explains from 10% to 47% of the phenotypic variance (listed as heritability denoted by *h* ^2^ in Table 1). Among the four SDL detected, all showed bias in favor of the XND126 parent, that is, the homozygote of XND126 allele was over represented at the cost of low representation of the other parent. The largest SDL locus is located in chromosome five at position 32.11 cM. The frequency of the heterozygote was close to the Mendelian frequency of 0.5, but the homozygote of Gaocheng 8901 allele was almost wiped out. The estimated genotypic frequencies are plotted against the genome location as shown in Figure 2. The deviation from Mendelian segregation is quite obvious for chromosome five. 

One of the major theoretical contributions of this study is the development of the variance-covariance matrix of the estimated QTL-SDL parameters. The covariances between pairs of estimated parameters are not of interest, but the variances of the estimated parameters are important. We reported the standard errors for two selected loci, locus 4 (QTL) and locus 7 (SDL). These standard errors are listed in Table 2. The standard errors are the square roots of the variances obtained from the EM algorithm. The variance-covariance matrix of the estimated parameters takes the inverse of the information matrix. As a result, they are approximate and biased downwards (Louis 1982). The approximation is close to the true variance only in large samples. We also performed a bootstrap analysis (1000 bootstrap samples) to provide more accurate estimation of the variance. The results of the bootstrap estimates of the standard errors for the two loci are also listed in Table 2 for comparison. The approximate standard errors from the EM algorithm are indeed biased downward, especially for locus 4 (QTL). The approximation is much better for locus 7 (SDL). In practice, the bootstraps method is recommended for obtaining more accurate estimates of the standard errors if the sample size is small.

## 4. Discussion

Statistical methods for mapping quantitative trait loci are well developed for Mendelian populations. Methods also are available for mapping viability loci or segregation distortion loci when markers do not segregate in a typical Mendelian ratio [4–6, 9–11]. However, QTL mapping and SDL mapping have never been combined in a single analysis. This study is the first attempt to combine the two seemingly different analyses into a joint one. When QTL and SDL are loosely linked or not linked, the joint analysis does not offer much advantage over the separate analyses. When they do overlap, a phenomenon called pleiotropy, joint mapping does offer some advantage. Unfortunately, the wheat experiment introduced here is not a good example to demonstrate the advantage of joint analysis because the QTL and SDL detected do not overlap. 

An obvious situation where the joint analysis can be more powerful is QTL mapping with selective genotyping. In most designed selective genotyping experiments, two groups of extreme phenotypes are selected for genotyping. The power increase under selective genotyping has been demonstrated [16]. Directional (one-tailed) selection is rarely used for selective genotyping because it artificially reduces the variation of the trait and thus reduces the statistical power of QTL detection. However, with the joint analysis, the power can increase under directional selection. Such a directional selection is common in breeding populations. We now use a simulated example to demonstrate this power increase. We simulated 500 F_2_ individuals for a single chromosome of 300 cM long. We placed 16 markers evenly over the chromosome with 20 cM per marker interval. A QTL was placed at position 150 cM of the chromosome. The QTL explains 5% of the phenotypic variance with additive effect only. This QTL is considered small or modest. We selected 300 smallest individuals out of the 500 (one-tailed selection) for mapping. The LOD test statistic profiles are depicted in Figure 3. Figure 3(a) shows the test statistic for QTL regardless whether segregation is distorted or not. The panel in the middle (b) shows the LOD test statistic for segregation distortion, regardless whether a QTL is present or not. The panel at the bottom (c) shows the LOD score profile of the joint analysis where the null model is no QTL and no SDL. If LOD = 3 is the threshold value, neither of the separate analyses is significant. However, the joint analysis has a LOD score as high as 4.5, indicating a significant QTL in the middle of the chromosome. This example clearly demonstrated the advantage of joint analysis over separate analyses. 

Segregation distortion may be caused by gametic selection, zygotic selection, or both. Our model was developed under zygotic selection because we are dealing with the genotypic frequencies. However, if the true cause of segregation is gametic selection, we can still detect segregation distortion as long as the gametic selection leads to the genotypic frequencies deviating from the expected Mendelian ratio. A model particularly handling gametic selection has not been developed yet, but it is not difficult. Similar to the zygotic selection model, gametic selection requires known marker linkage phases. Let us take the F_2_ population as an example to show the gametic selection model. Denote the frequencies of *G*_1_ and *G*_2_ alleles from the female parent by *ν*_1_^*f*^ and *ν*_2_^*f*^, respectively, for *ν*_1_^*f*^ + *ν*_2_^*f*^ = 1, and the corresponding allele frequencies from the male parent. by *ν*_1_^*m*^ and *ν*_2_^*m*^ for *ν*_1_^*m*^ + *ν*_2_^*m*^ = 1. Under Mendelian segregation, *ν*_1_^*f*^ = *ν*_2_^*f*^ = *ν*_1_^*m*^ = *ν*_2_^*m*^ = 1/2. When gametic selection occurs, we treat *ν*_1_^*f*^ and *ν*_1_^*m*^ as unknown parameters for estimation. The genotypic frequencies are simply functions of the two unknown parameters, as given by *ω*_1_ = *ν*_1_^*f*^ *ν*_1_^*m*^, *ω*_2_ = *ν*_1_^*f*^ *ν*_2_^*m*^ + *ν*_2_^*f*^ *ν*_1_^*m*^ and *ω*_3_ = *ν*_2_^*f*^ *ν*_2_^*m*^. QTL parameters are estimated using the same algorithm as described in the zygotic selection model because they depend only on the genotypic frequencies. Estimating parameters *ν*_1_^*f*^ and *ν*_1_^*m*^ requires a modified algorithm. Under the gametic selection model, we can test whether the segregation distortion is caused by the distortion of female gametes, male gametes, or both. This is an interesting topic that deserves further investigation. 

The joint analysis developed in this study only applies to line crossing data where the marker linkage phases are known. It cannot be applied to pedigree data analysis. Application of the method to pedigrees warrants further investigation and it is not obvious to us at this moment. However, extension to other line crossing families is possible. We have already extended the method to BC (backcrosses), RIL (recombinant inbred lines), DH (double haploids), and FW (four way crosses) and incorporated them into our QTL mapping program that is described in the next paragraph. The extension also includes dominance markers and missing marker genotypes. 

The proposed joint mapping applies to interval mapping only. Extension to multiple QTL/SDL mapping is difficult. However, interval mapping is still the quickest method of QTL mapping, even though multiple QTL mapping programs are available. Compared with traditional QTL interval mapping, this joint analysis involves one additional step of updating the genotypic frequencies. This additional step presents a complication where the conditional genotypic frequencies given flanking marker genotypes cannot be calculated prior to QTL mapping. They must be calculated with the phenotypic values along with the flanking marker genotypes. This complication makes modification of existing QTL mapping programs difficult. Fortunately, we have incorporated the joint QTL/SDL mapping into our QTL mapping program. This program is a SAS procedure called PROC QTL [17]. The METHOD = “ML” option in the PROC QTL statement must be turned on with an additional suboption /DISTORTION to invoke the joint mapping procedure. Without the /DISTORTION option, the ML analysis simply assumes Mendelian segregation. PROC QTL is available on our website (http://www.statgen.ucr.edu/software.html) and users can download the program with no charge. The program is also accompanied with a detailed user manual.

## Figures and Tables

**Table 1:** Estimated parameters for eight loci (QTL/SDL) of the wheat QTL and SDL analysis using an F_2_ family derived from two inbred lines of the wheat.

Locus	1	2	3	4	5	6	7	8
Type	SDL	QTL	QTL	QTL	QTL	SDL	SDL	SDL
Chromosome	1	1	2	2	2	3	5	5
Position (cM)	0.00	19.8	15.78	29.57	34.79	67.32	32.11	79.79
Interval^a^	0.00–5.45	0.00–35.15	12.40–18.18	27.41–31.74	34.35–36.10	59.21–67.32	22.00–35.25	72.56–102.84
LOD score	3.5	3.13	15.77	29.87	21.86	3.35	18.12	8.6
*ω*_1_	0.2099	0.1986	0.2334	0.2684	0.2637	0.1852	0.0763	0.1071
*ω*_2_	0.4239	0.5083	0.5456	0.5054	0.494	0.4568	0.4291	0.4801
*ω*_3_	0.3663	0.2931	0.221	0.2262	0.2423	0.358	0.4946	0.4128
*a*	0.1435	0.2076	0.3856	0.4438	0.3963	0.0307	−0.0539	0.0882
*d*	0.0494	0.1594	0.3071	0.4159	0.3542	0.0269	−0.0036	−0.2086
*σ* ^2^	0.2796	0.2622	0.2032	0.16	0.1847	0.2917	0.2913	0.2837
*h* ^2^	0.0375	0.0962	0.3252	0.4697	0.373	0.0022	0.005	0.0495
*β*	1.1247	1.0619	0.9509	0.8943	0.9398	1.1163	1.1023	1.2503

^a^Interval means one LOD drop supporting interval.

**Table 2:** Standard errors of the estimated parameters for loci 4 (QTL) and 7 (SDL) of the wheat F_2_ mapping population (see Table 1 for detailed information about loci 4 and 7). The StdErr (EM) and StdErr (Boots) represent the standard errors obtained from the EM algorithm and the bootstrap method, respectively.

Parameter	Locus 4(QTL)			Locus 7(SDL)		
	Estimate	StdErr (EM)	StdErr (Boots)	Estimate	StdErr (EM)	StdErr (Boots)
*β*	0.8943	0.03705	0.06233	1.1023	0.06934	0.07434
*a*	0.4438	0.03721	0.07086	−0.0539	0.06934	0.07653
*d*	0.4159	0.05258	0.08066	−0.0036	0.09138	0.09763
*σ* ^2^	0.1600	0.01515	0.04082	0.2913	0.02644	0.02771
*ω*_1_	0.2684	0.02905	0.02857	0.0763	0.01767	0.01749
*ω*_2_	0.5054	0.03274	0.03437	0.4291	0.03336	0.03511

## Appendices

### A. Standard Errors of the Estimated Parameters

Let us define the individual-wise complete-data log likelihood for plant *j* as

(A.1) $$\begin{array}{cc} & L_{j}\left(\theta ,\delta \right)\\ & =\left[-\frac{1}{2}\ln\;\left(\sigma^{2}\right)-\frac{1}{2\sigma^{2}}\overset{3}{\underset{\kappa =1}{\sum }}\delta \left(G_{j},\kappa \right){\left(y_{j}-X_{j}\beta -H_{\kappa }\gamma \right)}^{2}\right]\\ & +\overset{3}{\underset{\kappa =1}{\sum }}\delta \left(G_{j},\kappa \right)\left[\ln\;\left(T_{1}\left(\kappa ,M_{j}\right)\right)+\ln\;\left(T_{2}\left(\kappa ,N_{j}\right)\right)\right]\\ & +\overset{3}{\underset{\kappa =1}{\sum }}\delta \left(G_{j},\kappa \right)\ln\;\omega_{\kappa }, \end{array}$$

where *ω*_3_ = 1 − *ω*_1_ − *ω*_2_ so that *ω*_3_ is excluded from the parameter vector. To derive the variance covariance matrix of the estimated parameters, we need to define the score vector *S*_*j*_(*θ*, *δ*) and the Hessian matrix *H*_*j*_(*θ*, *δ*) for the individual-wise complete-data log likelihood function. The Louis [18] information matrix of the parameters under the EM algorithm is

(A.2) $$I\left(\hat{\theta }\right)=-\overset{n}{\underset{j=1}{\sum }}E\left[H_{j}\left(\hat{\theta },\delta \right)\right]-\overset{n}{\underset{j=1}{\sum }}\text{var}\left[S_{j}\left(\hat{\theta },\delta \right)\right],$$

where the expectation and variance are taken with respect to the missing values *δ*. Once the information matrix is define, the variance matrix of the estimated parameters simply takes

(A.3) $$\text{var}\left(\hat{\theta }\right)\approx I^{-1}\left(\hat{\theta }\right).$$

The standard error of each parameter takes the square root of each diagonal element of the above matrix. The approximation performs better for large sample sizes.

We now present the score vector and the Hessian matrix. The score vector is denoted by *S*_*j*_(*θ*, *δ*) = ∂*L*_*j*_(*θ*, *δ*)/∂*θ*, which consists of five blocks, as follows:

(A.4) $$\begin{array}{cc} S_{j}\left(\beta ,\delta \right) & =\frac{\partial L_{j}\left(\theta ,\delta \right)}{\partial \beta }=\frac{1}{\sigma^{2}}\overset{3}{\underset{k=1}{\sum }}\delta \left(G_{j},\kappa \right)X_{j}^{T}\left(y_{j}-X_{j}\beta -H_{\kappa }\gamma \right),\\ S_{j}\left(\gamma ,\delta \right) & =\frac{\partial L_{j}\left(\theta ,\delta \right)}{\partial \gamma }=\frac{1}{\sigma^{2}}\overset{3}{\underset{\kappa =1}{\sum }}\delta \left(G_{j},\kappa \right)H_{\kappa }^{T}\left(y_{j}-X_{j}\beta -H_{\kappa }\gamma \right),\\ S_{j}\left(\sigma^{2},\delta \right) & =\frac{\partial L_{j}\left(\theta ,\delta \right)}{\partial \sigma^{2}}=-\frac{1}{2\sigma^{2}}\\ & \;+\frac{1}{2\sigma^{4}}\overset{3}{\underset{\kappa =1}{\sum }}\delta \left(G_{j},\kappa \right){\left(y_{j}-X_{j}\beta -H_{\kappa }\gamma \right)}^{2},\\ S_{j}\left(\omega_{1},\delta \right) & =\frac{\partial L_{j}\left(\theta ,\delta \right)}{\partial \omega_{1}}=\delta \left(G_{j},1\right)\frac{1}{\omega_{1}}-\delta \left(G_{j},3\right)\frac{1}{1-\omega_{1}-\omega_{2}},\\ S_{j}\left(\omega_{2},\delta \right) & =\frac{\partial L_{j}\left(\theta ,\delta \right)}{\partial \omega_{2}}=\delta \left(G_{j},2\right)\frac{1}{\omega_{2}}-\delta \left(G_{j},3\right)\frac{1}{1-\omega_{1}-\omega_{2}}. \end{array}$$

Concatenating the above five scores vertically, we can get the score vector:

(A.5) $$S_{j}\left(\theta ,\delta \right)=\left[\begin{array}{c} S_{j}^{}\left(\beta ,\delta \right)\\ S_{j}^{}\left(\gamma ,\delta \right)\\ S_{j}^{}\left(\sigma^{2},\delta \right)\\ S_{j}^{}\left(\omega_{1},\delta \right)\\ S_{j}^{}\left(\omega_{2},\delta \right) \end{array}\right].$$

The Hessian matrix is denoted by *H*_*j*_(*θ*, *δ*) = ∂^2^ *L*_*j*_(*θ*, *δ*)/∂*θ*∂*θ* ^*T*^, which is block diagonal with non-zero blocks given as follows:

(A.6) $$\begin{array}{cc} H_{j}\left(\beta \beta \right) & =\frac{\partial^{2}L_{j}\left(\theta ,\delta \right)}{\partial \beta \partial \beta^{T}}=-\frac{1}{\sigma^{2}}X_{j}^{T}X_{j},\\ H_{j}\left(\beta \gamma \right) & =\frac{\partial^{2}L_{j}\left(\theta ,\delta \right)}{\partial \beta \partial \gamma^{T}}=-\frac{1}{\sigma^{2}}\overset{3}{\underset{\kappa =1}{\sum }}\delta \left(G_{j},\kappa \right)X_{j}^{T}H_{\kappa },\\ H_{j}\left(\beta \sigma^{2}\right) & =\frac{\partial^{2}L_{j}\left(\theta ,\delta \right)}{\partial \beta \partial \sigma^{2}}\\ & =-\frac{1}{\sigma^{4}}\overset{3}{\underset{\kappa =1}{\sum }}\delta \left(G_{j},\kappa \right)X_{j}^{T}\left(y_{j}-X_{j}\beta -H_{\kappa }\gamma \right),\\ H_{j}\left(\gamma \gamma \right) & =\frac{\partial^{2}L_{j}\left(\theta ,\delta \right)}{\partial \gamma \partial \gamma^{T}}=-\frac{1}{\sigma^{2}}\overset{3}{\underset{\kappa =1}{\sum }}\delta \left(G_{j},\kappa \right)H_{\kappa }^{T}H_{\kappa }^{},\\ H_{j}\left(\gamma \sigma^{2}\right) & =\frac{\partial^{2}L_{j}\left(\theta ,\delta \right)}{\partial \gamma \partial \sigma^{2}}\\ & =-\frac{1}{\sigma^{4}}\overset{3}{\underset{\kappa =1}{\sum }}\delta \left(G_{j},\kappa \right)H_{\kappa }^{T}\left(y_{j}-X_{j}\beta -H_{\kappa }^{}\gamma \right),\\ H_{j}\left(\sigma^{2}\sigma^{2}\right) & =\frac{\partial^{2}L_{j}\left(\theta ,\delta \right)}{\partial \sigma^{2}\partial \sigma^{2}}\\ & =\frac{1}{2\sigma^{4}}-\frac{1}{\sigma^{6}}\overset{3}{\underset{\kappa =1}{\sum }}\delta \left(G_{j},\kappa \right){\left(y_{j}-X_{j}\beta -H_{\kappa }\gamma \right)}^{2},\\ H_{j}\left(\omega_{1}\omega_{1}\right) & =\frac{\partial^{2}L_{j}\left(\theta ,\delta \right)}{\partial \omega_{1}\partial \omega_{1}}=-\delta \left(G_{j},1\right)\frac{1}{\omega_{1}^{2}}-\delta \left(G_{j},3\right)\frac{1}{\omega_{3}^{2}},\\ H_{j}\left(\omega_{1}\omega_{2}\right) & =\frac{\partial^{2}L_{j}\left(\theta ,\delta \right)}{\partial \omega_{1}\partial \omega_{2}}=-\delta \left(G_{j},3\right)\frac{1}{\omega_{3}^{2}},\\ H_{j}\left(\omega_{2}\omega_{2}\right) & =\frac{\partial^{2}L_{j}\left(\theta ,\delta \right)}{\partial \omega_{2}\partial \omega_{2}}=-\delta \left(G_{j},2\right)\frac{1}{\omega_{2}^{2}}-\delta \left(G_{j},3\right)\frac{1}{\omega_{3}^{2}}. \end{array}$$

The Hessian matrix is obtained through

(A.7) $$\begin{array}{cc} & H_{j}\left(\theta ,\delta \right)\\ & =\left[\begin{array}{ccccc} H_{j}\left(\beta \beta \right) & H_{j}\left(\beta \gamma \right) & H_{j}\left(\beta \sigma^{2}\right) & 0 & 0\\ H_{j}^{T}\left(\beta \gamma \right) & H_{j}\left(\gamma \gamma \right) & H_{j}\left(\gamma \sigma^{2}\right) & 0 & 0\\ H_{j}^{T}\left(\beta \sigma^{2}\right) & H_{j}^{T}\left(\gamma \sigma^{2}\right) & H_{j}^{}\left(\sigma^{2}\sigma^{2}\right) & 0 & 0\\ 0 & 0 & 0 & H_{j}^{}\left(\omega_{1}\omega_{1}\right) & H_{j}^{}\left(\omega_{1}\omega_{2}\right)\\ 0 & 0 & 0 & H_{j}^{T}\left(\omega_{1}\omega_{2}\right) & H_{j}^{}\left(\omega_{2}\omega_{2}\right) \end{array}\right] \end{array}.$$

The expectation of the Hessian matrix *E*[*H*_*j*_(*θ*, *δ*)]and the variance matrix of the score vector var[*S*_*j*_(*θ*, *δ*)] can be expressed explicitly because both the Hessian matrix and the score vector are linear functions of the missing value

(A.8) $$\delta_{j}={\left[\begin{array}{ccc} \delta (G_{j},1) & \delta (G_{j},2) & \delta (G_{j},3) \end{array}\right]}^{T}.$$

Therefore, *E*[*H*_*j*_(*θ*, *δ*)] and var[*S*_*j*_(*θ*, *δ*)] can eventually be expressed as functions of the expectation and variance of *δ*_*j*_, which have simple expressions because *δ*_*j*_ is a multinomial variable. The Hessian matrix *H*_*j*_(*θ*, *δ*) is already expressed in linear function of *δ*_*j*_ and thus the expectation can be obtained straightforwardly by replacing *δ*_*j*_ by *E*(*δ*_*j*_). An explicit linearity for the score function is not obvious. The following gives the linear relationship using matrix notation. Let us define the following matrices:

(A.9) $$\begin{array}{cc} C_{j}\left(\beta \right) & =\left[\begin{array}{c} \frac{1}{\sigma^{2}}X_{j}^{T}\left(y_{j}-X_{j}\beta -H_{1}\gamma \right)\\ \frac{1}{\sigma^{2}}X_{j}^{T}\left(y_{j}-X_{j}\beta -H_{2}\gamma \right)\\ \frac{1}{\sigma^{2}}X_{j}^{T}\left(y_{j}-X_{j}\beta -H_{3}\gamma \right) \end{array}\right],\\ C_{j}\left(\gamma \right) & =\left[\begin{array}{c} \frac{1}{\sigma^{2}}H_{1}^{T}\left(y_{j}-X_{j}\beta -H_{1}\gamma \right)\\ \frac{1}{\sigma^{2}}H_{2}^{T}\left(y_{j}-X_{j}\beta -H_{2}\gamma \right)\\ \frac{1}{\sigma^{2}}H_{3}^{T}\left(y_{j}-X_{j}\beta -H_{3}\gamma \right) \end{array}\right],\\ C_{j}\left(\sigma^{2}\right) & =\left[\begin{array}{c} \frac{1}{2\sigma^{4}}{\left(y_{j}-X_{j}\beta -H_{1}\gamma \right)}^{2}\\ \frac{1}{2\sigma^{4}}{\left(y_{j}-X_{j}\beta -H_{2}\right)}^{2}\\ \frac{1}{2\sigma^{4}}{\left(y_{j}-X_{j}\beta -H_{3}\right)}^{2} \end{array}\right],\\ C_{j}\left(\omega_{1}\right) & =\left[\begin{array}{c} \frac{1}{\omega_{1}}\\ 0\\ -\frac{1}{\omega_{3}} \end{array}\right],\;\;C_{j}\left(\omega_{2}\right)=\left[\begin{array}{c} 0\\ \frac{1}{\omega_{2}}\\ -\frac{1}{\omega_{3}} \end{array}\right]. \end{array}$$

The score vector in matrix notation is

(A.10) $$S_{j}\left(\theta ,\delta \right)=\frac{\partial L_{j}\left(\theta ,\delta \right)}{\partial \theta }=\left[\begin{array}{c} C_{j}^{T}\left(\beta \right)\\ C_{j}^{T}\left(\gamma \right)\\ C_{j}^{T}\left(\sigma^{2}\right)\\ C_{j}^{T}\left(\omega_{1}\right)\\ C_{j}^{T}\left(\omega_{2}\right) \end{array}\right]\delta_{j}+\left[\begin{array}{c} 0\\ 0\\ -\frac{1}{2\sigma^{2}}\\ 0\\ 0 \end{array}\right]=C_{j}\delta_{j}+D.$$

As a result,

(A.11) $$\text{var}\left[S_{j}\left(\theta ,\delta \right)\right]=C_{j}\text{var}\left(\delta_{j}\right)C_{j}^{T},$$

where

(A.12) $$\text{var}\left(\delta_{j}\right)=\mathrm{diag}\;\left[E\left(\delta_{j}\right)\right]-E\left(\delta_{j}\right)E\left(\delta_{j}^{T}\right)$$

is the variance-covariance matrix of the multinomial variable *δ*_*j*_.

### B. Estimation of Parameters under the Null Models

This appendix provides methods for estimating parameters under various null models that are required for constructing likelihood ratio test statistics.

#### B.1. Null Model for Testing QTL

The log likelihood for the null model is

(B.1) $$L_{S}\left(0,\hat{\omega }\right)=L\left(\hat{\beta },{\hat{\sigma }}^{2}\;|\;y\right)+L\left(\hat{\omega }m\right),$$

where

(B.2) $$\begin{array}{cc} L\left(\hat{\beta },{\hat{\sigma }}^{2}\;|\;y\right) & =-\frac{n}{2}\ln\;\left({\hat{\sigma }}^{2}\right)-\frac{1}{2{\hat{\sigma }}^{2}}\overset{n}{\underset{j=1}{\sum }}{\left(y_{j}-X_{j}\hat{\beta }\right)}^{2},\\ L\left(\hat{\omega }\;|\;m\right) & =\overset{n}{\underset{j=1}{\sum }}\ln\;\left[\overset{3}{\underset{\kappa =1}{\sum }}{\hat{\omega }}_{\kappa }\Gamma_{1}\left(\kappa ,M_{j}\right)\Gamma_{2}\left(\kappa ,N_{j}\right)\right]. \end{array}$$

The MLEs of the population parameters are obtained explicitly without iteration:

(B.3) $$\begin{array}{cc} \hat{\beta } & ={\left[\overset{n}{\underset{j=1}{\sum }}X_{j}^{T}X_{j}^{}\right]}^{-1}\left[\overset{n}{\underset{j=1}{\sum }}X_{j}^{T}y_{j}\right],\\ {\hat{\sigma }}^{2} & =\frac{1}{n}\overset{n}{\underset{j=1}{\sum }}{\left(y_{j}-X_{j}\hat{\beta }\right)}^{2}. \end{array}$$

The estimated genotypic frequencies, however, require the following iterations:

(B.4) $$\omega_{\kappa }^{\left(t+1\right)}=\frac{1}{n}\overset{n}{\underset{j=1}{\sum }}E\left[\delta \left(G_{j},\kappa \right)\right],\;\forall \kappa =1,2,3,$$

where

(B.5) $$E\left[\delta \left(G_{j},\kappa \right)\right]=\frac{\omega_{\kappa }^{\left(t\right)}\Gamma_{1}\left(\kappa ,M_{j}\right)\Gamma_{2}\left(\kappa ,N_{j}\right)}{\sum_{\xi =1}^{3}\omega_{\xi }^{\left(t\right)}\Gamma_{1}\left(\xi ,M_{j}\right)\Gamma_{2}\left(\xi ,N_{j}\right)}.$$

#### B.2. Null Model for Testing SDL

The log likelihood function for the null model is

(B.6) $$\begin{array}{cc} & L_{Q}\left(\hat{\gamma },\phi \right)\\ & =-\frac{n}{2}\ln\;\left({\hat{\sigma }}^{2}\right)\\ & +\overset{n}{\underset{j=1}{\sum }}\ln\;\left\{\overset{3}{\underset{\kappa =1}{\sum }}\phi_{\kappa }\exp\;\left[-\frac{1}{2{\hat{\sigma }}^{2}}{\left(y_{j}-{\hat{\mu }}_{\kappa }\right)}^{2}\right]\Gamma_{1}\left(\kappa ,M_{j}\right)\Gamma_{2}\left(\kappa ,N_{j}\right)\right\}. \end{array}$$

Finding the solution of the parameters requires iterations as given as foolows:

(B.7) $$\begin{array}{cc} \beta^{\left(t+1\right)} & ={\left[\overset{n}{\underset{j=1}{\sum }}X_{j}^{T}X_{j}^{}\right]}^{-1}\\ & \;\times \left[\overset{n}{\underset{j=1}{\sum }}\;\overset{3}{\underset{\kappa =1}{\sum }}E\left[\delta \left(G_{j},\kappa \right)\right]X_{j}^{T}\left(y_{j}-H_{\kappa }\gamma^{\left(t\right)}\right)\right],\\ \gamma^{\left(t+1\right)} & ={\left[\overset{n}{\underset{j=1}{\sum }}\;\overset{3}{\underset{\kappa =1}{\sum }}E\left[\delta (G_{j},\kappa )\right]\left(H_{\kappa }^{T}H_{\kappa }^{}\right)\right]}^{-1}\\ & \;\times \left[\overset{n}{\underset{j=1}{\sum }}\;\overset{3}{\underset{\kappa =1}{\sum }}E\left[\delta \left(G_{j},\kappa \right)\right]H_{\kappa }^{T}\left(y_{j}-X_{j}\beta^{\left(t\right)}\right)\right],\\ \sigma^{2(t+1)} & =\frac{1}{n}\overset{n}{\underset{j=1}{\sum }}\;\overset{3}{\underset{\kappa =1}{\sum }}E\left[\delta \left(G_{j},\kappa \right)\right]{\left(y_{j}-X_{j}\beta^{\left(t\right)}-H_{\kappa }\gamma^{\left(t\right)}\right)}^{2}, \end{array}$$

where

(B.8) $$\begin{array}{cc} & E\left[\delta \left(G_{j},\kappa \right)\right]\\ & =\frac{\phi_{\kappa }\exp\;\left[-\left(1/2\sigma^{2}\right){\left(y_{j}-\mu_{\kappa }\right)}^{2}\right]\Gamma_{1}\left(\kappa ,M_{j}\right)\Gamma_{2}\left(\kappa ,N_{j}\right)}{\sum_{\xi =1}^{3}\phi_{\xi }\exp\;\left[-\left(1/2\sigma^{2}\right){\left(y_{j}-\mu_{\xi }\right)}^{2}\right]\Gamma_{1}\left(\xi ,M_{j}\right)\Gamma_{2}\left(\xi ,N_{j}\right)}. \end{array}$$

The Mendelian frequencies *ϕ*_*κ*_'s are constants, not parameters, as *ϕ*_1_ = *ϕ*_3_ = (1/2)*ϕ*_2_ = 1/4.

#### B.3. Null Model for Testing Both QTL and SDL

The log likelihood function for the null model is

(B.9) $$L\left(0,\phi \right)=L\left(\hat{\beta },{\hat{\sigma }}^{2}\;|\;y\right)+L\left(\phi \;|\;m\right),$$

where

(B.10) $$\begin{array}{cc} & L\left(\hat{\beta },{\hat{\sigma }}^{2}\;|\;y\right)=-\frac{n}{2}\ln\;\left({\hat{\sigma }}^{2}\right)-\frac{1}{2{\hat{\sigma }}^{2}}\overset{n}{\underset{j=1}{\sum }}{\left(y_{j}-X_{j}\hat{\beta }\right)}^{2},\\ & L\left(\phi m\right)=\overset{n}{\underset{j=1}{\sum }}\ln\;\left[\overset{3}{\underset{\kappa =1}{\sum }}\phi_{\kappa }\Gamma_{1}\left(\kappa ,M_{j}\right)\Gamma_{2}\left(\kappa ,N_{j}\right)\right]. \end{array}$$

The MLEs of parameters are

(B.11) $$\begin{array}{cc} & \hat{\beta }={\left[\overset{n}{\underset{j=1}{\sum }}X_{j}^{T}X_{j}^{}\right]}^{-1}\left[\overset{n}{\underset{j=1}{\sum }}X_{j}^{T}y_{j}\right],\\ & {\hat{\sigma }}^{2}=\frac{1}{n}\overset{n}{\underset{j=1}{\sum }}{\left(y_{j}-X_{j}\hat{\beta }\right)}^{2}. \end{array}$$

They are the same as those provided in (B.3). The Mendelian frequencies *ϕ*_*κ*_'s are constants, not parameters.

## References

[1] Xu S (2008). Quantitative trait locus mapping can benefit from segregation distortion. *Genetics*.

[2] Faris JD, Laddomada B, Gill BS (1998). Molecular mapping of segregation distortion loci in *Aegilops tauschii*. *Genetics*.

[3] Kärkkäinen K, Koski V, Savolainen O (1996). Geographical variation in the inbreeding depression of scots pine. *Evolution*.

[4] Luo L, Xu S (2003). Mapping viability loci using molecular markers. *Heredity*.

[5] Vogl C, Xu S (2000). Multipoint mapping of viability and segregation distorting loci using molecular markers. *Genetics*.

[6] Fu YB, Ritland K (1994). On estimating the linkage of marker genes to viability genes controlling inbreeding depression. *Theoretical and Applied Genetics*.

[7] Lorieux M, Perrier X, Goffinet B, Lanaud C, de León DG (1995). Maximum-likelihood models for mapping genetic markers showing segregation distortion. 2. F_2_ populations. *Theoretical and Applied Genetics*.

[8] Lorieux M, Goffinet B, Perrier X, de León DG, Lanaud C (1995). Maximum-likelihood models for mapping genetic markers showing segregation distortion. 1. Backcross populations. *Theoretical and Applied Genetics*.

[9] Wang C, Zhu C, Zhai H, Wan J (2005). Mapping segregation distortion loci and quantitative trait loci for spikelet sterility in rice (*Oryza sativa* L.). *Genetical Research*.

[10] Luo L, Zhang Y-M, Xu S (2005). A quantitative genetics model for viability selection. *Heredity*.

[11] Zhu C, Zhang Y-M (2007). An EM algorithm for mapping segregation distortion loci. *BMC Genetics*.

[12] Elston RC, Stewart J (1973). The analysis of quantitative traits for simple genetic models from parental, F_1_ and backcross data. *Genetics*.

[13] Dempster AP, Laird MN, Rubin DB (1977). Maximum likelihood from incomplete data via the *EM* algorithm. *Journal of the Royal Statistical Society, Series B*.

[14] Dou B, Hou B, Xu H (2009). Efficient mapping of a female sterile gene in wheat (*Triticum aestivum* L.). *Genetics Research*.

[15] Piepho H-P (2001). A quick method for computing approximate thresholds for quantitative trait loci detection. *Genetics*.

[16] Ronin YI, Korol AB, Weller JI (1998). Selective genotyping to detect quantitative trait loci affecting multiple traits: interval mapping analysis. *Theoretical and Applied Genetics*.

[17] Hu Z, Xu S PROC QTL—a SAS procedure for mapping quantitative trait loci.

[18] Louis TA (1982). Finding the observed information matrix when using the *EM* algorithm. *Journal of the Royal Statistical Society, Series B*.

